# Development of a time-resolved immunochromatographic strip for rapid and quantitative determination of deoxynivalenol

**DOI:** 10.3389/fvets.2023.1142820

**Published:** 2023-03-16

**Authors:** Jingneng Wang, Lihua Wang, Hui Zhang, Xinglin Mei, Liangzhu Qiu, Jing Liu, Yongsong Zhou

**Affiliations:** ^1^Shanghai Xiongtu Biotechnology Co., Ltd., Shanghai, China; ^2^Jiangsu Agri-Animal Husbandry Vocational College, Taizhou, China; ^3^Institute of Food Safety and Nutrition, Jiangsu Academy of Agricultural Sciences, Nanjing, China

**Keywords:** deoxynivalenol, time-resolved fluorescence immunoassay, quantitative analysis, rapid testing, food crop, animal feed

## Abstract

Deoxynivalenol (DON) contamination of food crops and feeds is almost impossible to avoid completely; however, through best management practices, this risk can be effectively managed and maximumly mitigated. Accurate and rapid detection of DON contamination as early in the entire value chain as possible is critical. To achieve this goal, we developed a DON test strip based on time-resolved fluorescence immunoassay (TRFIA) and a specific DON monoclonal antibody for the rapid quantification of DON in food crops and feeds. The strip displayed a good linearity (*R*^2^ = 0.9926), with a limit of quantification of 28.16 μg/kg, a wide linear range of 50 ~ 10,000 μg/kg. The intra-batch coefficient of variation (CV) and the inter-batch CV was <5.00 and 6.60%, respectively. This TRFIA-DON test strip was applied to detect DON in real samples, and the accuracy and reliability were confirmed by liquid chromatography-mass spectrometry (LC-MS/MS). Results showed that the relative standard deviation between the DON strips and LC-MS/MS was <9%. The recovery rates in corn samples ranged from 92 to 104%. The established TRFIA-DON test strip had the characteristics of high sensitivity, high accuracy, and a wide linear range which was suitable for rapid and quantitative determination of DON in food crops and feeds at both on-site and laboratory.

## 1. Introduction

Deoxynivalenol (DON) is one of the five most important mycotoxins in agriculture (1), and it is also the most widely distributed toxin with the highest contamination level among the trichothecenes (2). Although the toxicity of DON is relatively less than other mycotoxins, it remains a primary concern in many countries because DON can not only cause major economic losses to animal husbandry, but also seriously endanger human and animal health. DON is most harmful to pigs, followed by rodents, dogs, cats, poultry, and ruminants (3). Because DON is harmful to almost all animals, many countries have established the tolerable limit standard of DON in animal feed. The recommended levels of DON in animal feed are ranged from 0.9 to 12 mg/kg by regulatory public agencies worldwide (4). Mycotoxins such as DON are estimated to cause billions of dollars in economic losses in both China and the United States each year (5, 6). The USA, India and China have reported acute vomiting in humans due to ingest foods contaminated with DON (7). In the face of DON, the biggest problem is not that it is widespread in food crop and animal feed (8, 9), but that we have not found an effective method to mitigate it until now (10). In addition, the harmful effects are becoming worse and worse because of climate change (1, 11). DON contamination cannot be completely avoided (12), and there is no ideal detoxification strategy, therefore, it is particularly important to detect DON as early as possible.

Mycotoxin analytical methods can be artificially divided into three broad categories: immunobased, direct, and indirect (12). Of the three categories, antibody-based immunoassay such as enzyme-linked immunosorbent assay (13, 14), fluorescence polarization immunoassay (15, 16), and surface plasmon resonance (17) are probably the most commonly used method. The direct methods are usually the most accurate which include liquid chromatography-mass spectrometry (LC-MS/MS) (18), gas chromatography-mass spectrometry (19), and high-performance liquid chromatography (20, 21). Indirect testing methods, including near infrared spectroscopy (22), mid-infrared spectroscopy (23), and hyperspectral imaging (24), rely on other characteristics to infer the presence or absence of mycotoxins. They are not ideal methods when considered in terms of the speed of detection, the cost and performance. For example, direct methods are most precise and accurate, but also the most expensive; the speed and cost of gold immunochromatography assay (GICA) is acceptable, but it is not accurate enough.

Since the identification of DON in 1970's (25), researchers have been working hard to develop an accurate, rapid, and cost-effective testing method, however, the success remains elusive (1). Therefore, we try to develop a time-resolved immunochromatographic strip for rapid and quantitative determination of deoxynivalenol. Time-resolved fluorescence immunoassay (TRFIA) is a promising analytical method because of its high specificity, high sensitivity, and low cost. Recently, a lot of analytical methods based on TRFIA to detect DON, zearalenone, fumonisin, aflatoxin, ochratoxin A, and T-2 toxin in cereals and feed were developed and applied (6, 26–32). However, as far as DON detection is concerned, the existing TRFIA-based test strips cannot meet the needs of industrial applications either in terms of their sensitivity or accuracy.

In order to improve the sensitivity and accuracy of the test strip, we combined time-resolved fluorescence with immunochromatography and established a TRFIA strip for the rapid testing and quantification of DON. First, time-resolved fluorescent microspheres were used to label highly specific DON antibodies. Then, the key experimental parameters affecting the performance of the strip were optimized. Thirdly, the sensitivity, accuracy, specificity, and stability of the strip were evaluated. Finally, the performance of the TRFIA-DON strip was compared with that of LC-MS/MS. The TRFIA-DON strip developed in this study can be used for rapid and quantitative detection of DON contamination in food crops and feeds.

## 2. Materials and methods

### 2.1. Reagents and materials

Bovine serum albumin (BSA), N-hydroxysuccinimide (NHS, 0.5 mg/mL), and 1-(3-dimethylaminopropyl)-3-ethylcarbonimide hydrochloride (EDC, 0.5 mg/mL) were obtained from Sigma-Aldrich (St. Louis, Missouri, MO, USA). Methanol, sucrose, sodium chloride and other chemical reagents were purchased from Sinopharm Chemical Reagent Co., Ltd. (Shanghai China). Quality control material DON in maize was purchased from Academy of National Food and Strategic Reserves Administration (1,410 ± 110 μg/kg, GBW(E) 100383, Beijing China). DON solution was purchased from ANPEL Laboratory Technologies, Inc. (Shanghai China). Europium Chelate Microspheres and Fluoro-Max Fluorescent Carboxylate-Modified Particles (europium chelate PS-COOH; nominal diameter, 200 nm; m/v, 1%) were purchased from Bangs Laboratories, Inc. (Fishers, IN, USA) and Thermo Fisher Scientific, Inc. (Waltham, MA, USA), respectively. The DON monoclonal antibody, DON antigen, polyvinyl chloride (PVC) board, nitrocellulose (NC) membrane, absorbent pad, and glass cellulose membrane were purchased from Yangzhou Keneng Biotechnology Co., Ltd. (Yangzhou, Jiangsu, China). The solutions used to pretreat the conjugate pad, activate the microspheres (0.05 mol/L 2-(N-morpholino) ethanesulfonic acid, pH 6.0), dilute the microspheres (0.05 mol/L Tris, pH8.0, containing 10% sucrose, 0.05% Procline 300), and preserve the microsphere-antibody conjugate (0.05 mol/L Tris, pH8.0, containing 10% trehalose, 1% BSA, 0.05% Procline 300) were purchased from Shanghai Xiongtu Biotechnology Co., Ltd. (Shanghai China). The sample extracting solution is a mixture of acetonitrile, deionized water, and acetic acid (v/v, 70:29.5:0.5). The acetonitrile and acetic acid were purchased from Yangzhou Keneng Biotechnology.

### 2.2. Principal instruments

The portable TRFIA analyzer (XT8201A) and the thermostatic incubator (XT8202A) for test strips were purchased from Shanghai Xiongtu Biotechnology. The HGS510 dispense system, the guillotine cutter, ultrasonic instrument, and high-speed refrigerated centrifuge were purchased from Yangzhou Keneng Biotechnology. The LC-MS/MS instrument was manufactured by Waters Co., (Milford, MA, USA).

### 2.3. Labeling of mouse anti-DON antibody by fluorescent microspheres

Activation solution of microspheres (900 μL) and 100 μL of fluorescent microspheres were placed in a 2 mL centrifuge tube and mixed by ultrasound machine. Then 40 μL of NHS-EDC (v/v, 1:1) solution were added to the centrifuge tube and placed at room temperature for 40 min in a dark place. The obtained mixture was centrifuged at 14,000 r/min for 20 min to discard the supernatant. Subsequently, 500 μL of boric acid buffer and 50 μL of DON monoclonal antibody were added to the precipitate and vortexed for 1 min. The antibody and microsphere mixtures were incubated at room temperature for 2 h in a dark place. Next, 500 μL of blocking solution were added and reacted at room temperature for 60 min. The mixture was centrifuged at 14,000 r/min for 20 min to discard the supernatant. Finally, the precipitate was redissolved by adding 1 mL of microsphere-antibody conjugate preservation solution and stored at 4°C.

### 2.4. Assembly of TRFIA-DON test strip

**Figure 1:**
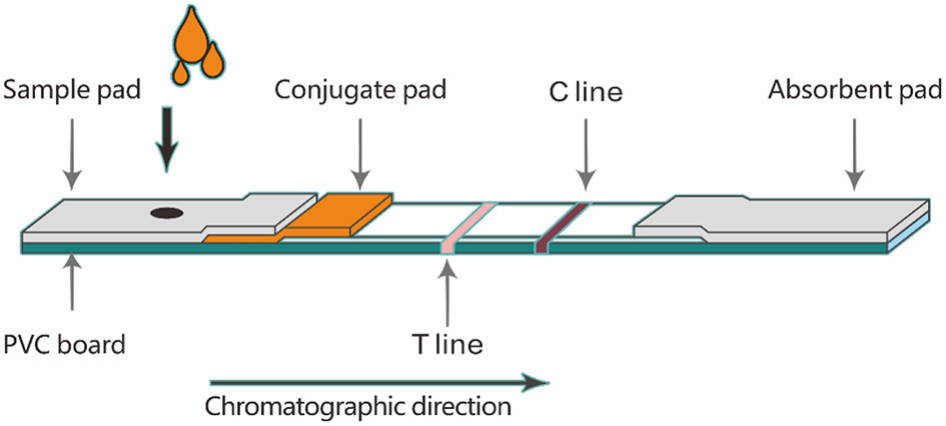
Assembly diagram of time-resolved fluorescence immunoassay test strip.

Firstly, an NC membrane was affixed to the PVC board. Then, 0.2 mg/mL goat anti-mouse IgG and 0.25 mg/mL DON antigen were sprayed onto the NC membrane as a C line and a T line, respectively. The NC-PVC board was placed in a drying oven to dry at 37°C overnight. The microsphere-antibody conjugate was diluted 40 times with diluent and sprayed on the glass cellulose membrane as a conjugate pad. The conjugate pad was placed in a drying oven to dry at 37°C overnight. As shown in Figure 1, the absorbent pad, conjugate pad, and sample pad were assembled in turn. After the assembly was completed, the assembled board was cut into a 4 mm wide strip with the guillotine cutter. The 4 mm wide strip was placed into a plastic card to produce a DON test strip. DON test strips were sealed in a dark plastic bag and stored at 4°C for further experiments.

### 2.5. Screening the optimal ratio of microspheres and antibodies

Two different brands of microspheres (100 μL each) were coupled with different amounts of DON monoclonal antibody. The ratios of microspheres to antibodies were 8:1, 4:1 and 1:1, respectively. Each microsphere-antibody conjugate was diluted 40 times and sprayed on the conjugate pad. When the conjugate pad was dried at 37°C overnight, assemble the TRFIA-DON test strip according to the process shown in Figure 1. To screen the optimal ratio of microspheres and antibodies, 100 μL of boric acid buffer (0.2 M, pH 7.5) were added to the sample well of TRFIA-DON test strip and detected by portable TRFIA analyzer. The test was repeated three times for each strip with different ratios of microspheres and antibodies. The optimal microsphere-antibody combination was selected by comparing the intensity and stability of the fluorescence signal values.

### 2.6. Screening of pretreatment solution for conjugate pad

Five different conjugate pad pre-treatment solutions (CPPS), numbered CPPS-A, CPPS-B, CPPS-C, CPPS-D and CPPS-E, were sprayed on five different glass cellulose membranes. The pre-treated glass cellulose membranes were dried at 37°C overnight, and then the microsphere-antibody conjugates were sprayed on them. The portable TRFIA analyzer was used to detect the fluorescence signal values of T and C lines for each test strip.

### 2.7. NC membrane drying time

Three NC membranes were taken and dried for 12, 24 and 48 h, respectively. These NC membranes were then used to assemble TRFIA-DON test strips. The fluorescence signal values of T and C lines of the sample with DON concentration of 0, 100, 1,000, 2,000, 5,000 and 10,000 μg/kg were determined and the T/C ratios were calculated.

### 2.8. Screening of microsphere diluents

Six different microsphere diluents (#1, #2, #3, #4, #5 and #6) were used to dilute the same microsphere-antibody conjugate, respectively. The volume ratio of diluent to microsphere-antibody conjugate was 40:1. The microsphere-antibody conjugates diluted with different diluents were sprayed on the conjugate pad, and then the TRFIA-DON test strips were prepared according to the steps shown in Figure 1. These strips were stored at 4 and 37 C for 21 days. The T-line and C-line fluorescence signal values of these strips were measured on day 0, day 3, day 6, day 9, day 12, day 15, day 18, and day 21, respectively.

### 2.9. Establishment of a standard curve

DON standard solution was prepared into working solution with concentrations of 20, 50, 100, 300, 700, 1,500, 3,000, 5,000, 8,000, and 12,000 μg/kg, respectively. Working solution (100 μL) and 600 μL of boric acid buffer (0.2 M, pH 7.5) were placed in a 2 mL centrifuge tube and thoroughly mixed. Then, 100 μL of mixtures were tested and recorded the T-line and C-line fluorescence signal values. Four Parameter Logistic Regression was used to draw the standard curve for quantitative determination of DON. The X-axis and Y-axis were represented by the natural logarithm of each working solution concentration and the ratio of T/C value corresponding to each concentration to T0/C0 value of 3.62 μg/kg a natural maize sample, respectively. The concentration of DON in the natural maize sample was determined by the LC-MS/MS method (GB 5009.111-2016) (33). First, the T and C values of DON standard solutions with different concentrations were measured with DON test strips, and the T/C values of each concentration point were calculated. Then, T and C values of a natural maize sample was measured with DON test strips, and T/C value of the natural maize sample was calculated, which was defined as T0/C0 value. Finally, the T/C value at each concentration point was divided by the T0/C0 value as the Y-axis value.

### 2.10. Sample pretreatment and testing

First, the sample to be tested was thoroughly mixed, and 1.00 g was placed in a 10 mL centrifuge tube, and then 5 mL sample extracting solutions were added to the centrifuge tube. The centrifuge tube was vortexed for 5 min, and centrifuged at 4,000 r/min for 2 min. Then, 100 μL of supernatant and 600 μL of boric acid buffer (0.2 M, pH 7.5) were taken and placed into 2 mL centrifuge tube. Subsequently, 100 μL of mixtures were dripped into the sample well of TRFIA-DON test strip. The TRFIA-DON test strip was placed on a thermostatic incubator and incubated at 37 °C for 6 min. After the incubation reaction was complete, the TRFIA-DON test strip was immediately inserted into the portable TRFIA analyzer. The fluorescence signal values of T-line and C-line were measured and the concentration of DON in sample was automatically calculated by the TRFIA instrument.

### 2.11. Determination of DON by LC-MS/MS

The concentration of DON in the sample was determined by the LC-MS/MS method. For details, refer to the National Standards of the People's Republic of China (GB/T 30956–2014, GB 5009.111-2016) (33, 34).

### 2.12. Evaluation the performance of TRFIA-DON test strip

#### 2.12.1. Sensitivity

A total of 20 negative samples were test by the prepared TRFIA-DON test strips, and then the mass concentration was calculated. The standard deviation (SD) and average (*X*) were obtained. The limit of quantification (LOQ) of the test strip was calculated as the following formula: LOQ = *X* + 10 SD.

#### 2.12.2. Precision

DON contamination samples were prepared by adding DON solution to negative corn samples, and negative corn samples were identified by national standard method (34). The concentrations of DON in the contaminated samples were 100,1,000 and 3,500 μg/kg, respectively. The contaminated samples were tested with strips from the same batch and three different batches. Each batch of test strips was repeated 6 times (using the same reagent and the same standard substance), and the coefficients of variation (CVs) within the same batch and between different batches were calculated to evaluate the precision of the strips.

#### 2.12.3. Accuracy

To evaluate the accuracy of the TRFIA-DON test strip, six pieces of quality control material DON in corn were weighed separately (1 g each). The DON was extracted using the method described in the section of “Sample pretreatment and testing.” The concentrations of DON in supernatant were detected with the prepared strips, and the recovery rates of the strips were calculated.

#### 2.12.4. Stability

The prepared TRFIA-DON test strips were encapsulated in aluminum foil bags and stored at 4°C for 90 days. The DON contamination samples with concentrations of 0, 100, 1,500, and 7,000 μg/kg were detected by randomly selecting test strips. Each concentration was repeated 6 times. The T-line and C-line fluorescence signal values of these strips were measured on day 1, day 15, day 30, day 45, day 60, day 75, and day 90, respectively.

#### 2.12.5. Applicability

Different types of crops and feeds, including wheat, corn, flour, poultry feed, laying hen feed, piglet feed, pig compound feed, sow feed, and supplemental feed for calf concentrates, 10 samples of each type, were randomly selected from the market and pretreated. All samples were detected by the LC-MS/MS method and the established TRFIA-DON test strip, respectively. The test was repeated three times for each sample.

## 3. Results

### 3.1. Optimal ratio of microspheres and antibodies

**Figure 2:**
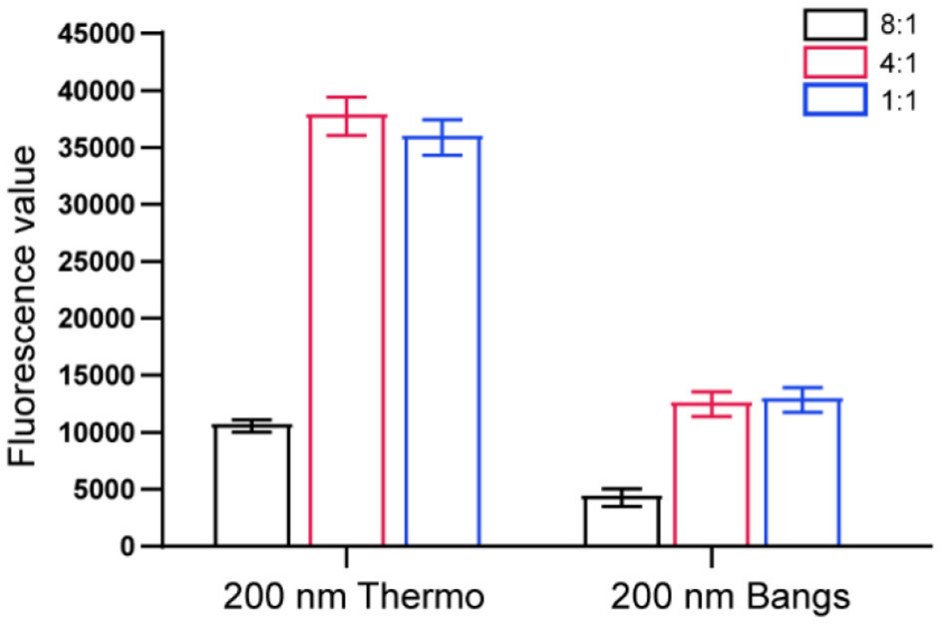
Screening the optimal ratio of microspheres and antibodies.

As shown in Figure 2, the fluorescence signal values of T-line and C-line increased as the ratio of microspheres to antibodies gradually decreased. When the ratio of microspheres to antibody was 4:1 and 1:1, ideal fluorescence signal intensity could be obtained. However, from the perspective of economy, 1:1 was finally selected as the ratio of microspheres to antibody coupling in this study. In addition, the same amount of antibody was used to conjugate different brands of microspheres. In this experiment, the fluorescence signal intensity of Fluoro-Max Fluorescent Carboxylate-Modified Particles (Thermo Fisher Scientific) was significantly higher than that of Europium Chelate Microspheres (Bangs Laboratories). Therefore, Thermo's fluorescent microspheres were selected in the follow-up experiment of this study.

### 3.2. Conjugate pad pretreatment solution

In order to select the most suitable conjugate pad for this experimental study, a total of five different pretreatment solutions were tested. The test was repeated three times for each pretreatment solution. The results showed that the strongest fluorescence signals were found in strips assembled from CPPS-D treated conjugate pads, indicating that CPPS-D had the best effect on the release of fluorescent microspheres (Table 1). CV values of CPPS-D and CPPS-E were the smallest among the five pretreatment solutions, all of which were 3%. The effects of different pretreatment solutions on fluorescence signal values were analyzed by analysis of variance. No significant difference was observed between pretreatment solutions (Table 2). Higher fluorescence signal intensity and lower CV predicted that the test strip would have a wider linear range and better stability. Therefore, the CPPS-D was selected as the pretreatment solution for conjugate pad.

**Table 1:** Effects of different pretreatment solutions on fluorescent microspheres.

**Fluorescence signal values (FSV)**	**CPPS-A**	**CPPS-B**	**CPPS-C**	**CPPS-D**	**CPPS-E**
FSV-1	5,210	5,749	4,267	6,197	5,519
FSV-2	6,083	5,286	4,523	6,061	5,486
FSV-3	5,751	6,085	4,956	6,435	5,258
Average	5,681	5,707	4,582	6,231	5,421
SD	441	401	348	189	142
CV (%)	8	7	8	3	3

**Table 2:** Significance test of five pretreatment solutions used analysis of variance.

	**CPPS-A**	**CPPS-B**	**CPPS-C**	**CPPS-D**	**CPPS-E**
CPPS-A	-	0.73	0.26	0.58	0.57
CPPS-B	0.37	-	0.23	0.13	0.73
CPPS-C	2.83	3.37	-	0.19	0.84
CPPS-D	0.72	6.95	4.23	-	0.93
CPPS-E	0.74	0.36	0.19	0.08	-

The F values are shown at the bottom left diagonal of the table. The P values are shown at the top right diagonal of the table.

### 3.3. NC membrane drying time

**Figure 3:**
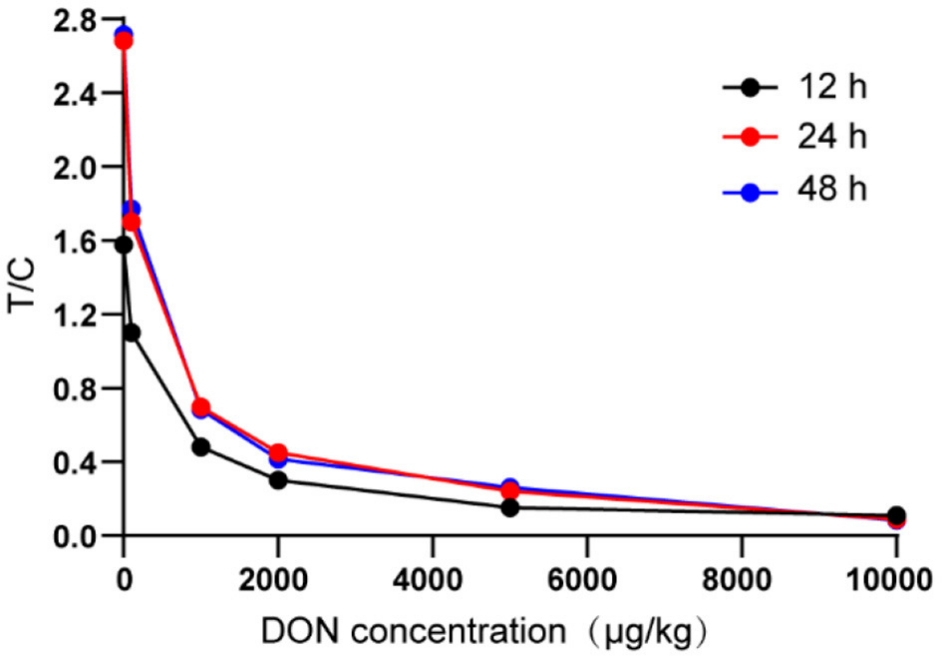
Determine the drying time of NC membrane.

It was not difficult to find from Figure 3 that compared with 12 h of drying, the T/C range of the test strip composed of 24 h of drying NC membrane was wider and more sensitivity, which was more conducive to the establishment of standard curve and the improvement of the accuracy of the test strip. In terms of sensitivity and linear range, there was no significant difference between the NC membrane dried for 48 h and that dried for 24 h.

### 3.4. Microsphere diluents

**Figure 4:**
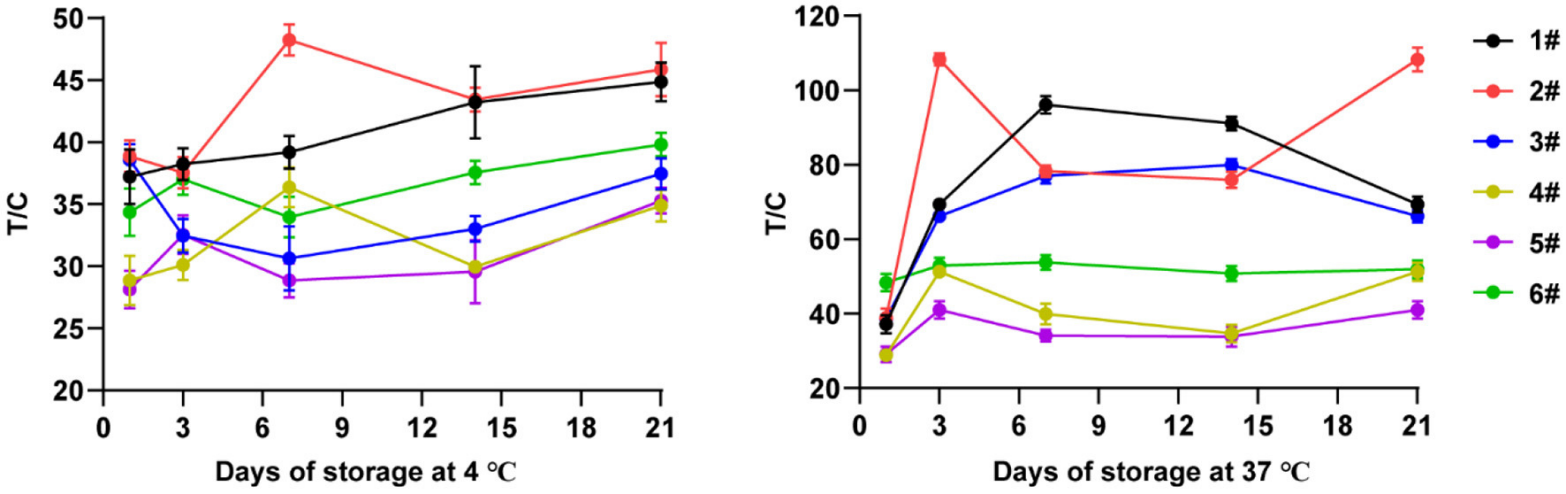
Influence of different microsphere diluents on the stability of test strips.

After 21 days of storage and regular sampling detection, the results were shown in Figure 4. It can be seen from the results that the stability of the test strip made by the diluent of No. 6 was the best whether it was stored at 4 or 37°C. Microsphere diluent No. 6 had the smallest SD value, which was 2.42 and 2.13 at 4 and 37°C storage conditions, respectively. Therefore, the No. 6 microsphere diluent was used in the follow-up experiment of this study.

### 3.5. Standard curve and linearity of the TRFIA-DON test strip

**Figure 5:**
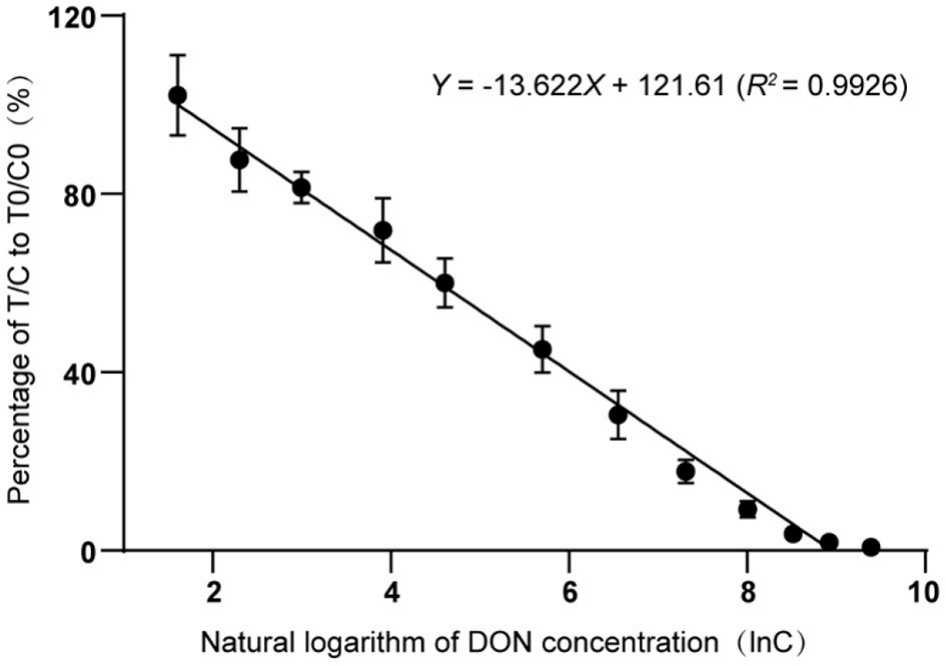
Standard curve of deoxynivalenol detection *via* time-resolved fluorescence immunoassay.

To establish a standard curve, the fluorescence signal intensities on T-line and the C-line with different DON concentrations were tested and recorded by a TRFIA instrument (Figure 5). Each concentration was measured and recorded three times. The standard curve exhibited good linearity when the natural logarithm ranged from 1.609 to 9.393, corresponding to concentrations ranging from 5 μg/kg to 12,000 μg/kg. In this concentration range, the inhibition rate ranged from 92 to 95%. The fitted linear regression equation was Y = −13.622X + 121.61 (R^2^ = 0.9926). From piecewise linear regression equation, we could deduce that the linear range of the curve was 50 ~ 10,000 μg/kg.

### 3.6. Performance evaluation

#### 3.6.1. Sensitivity

In order to verify the sensitivity of DON strips, we tested 20 negative samples with the established DON strips, and the results were shown in Table 3. According to the linear regression equation, the LOQ of the developed DON strips was calculated as 28.16 μg/kg.

**Table 3:** Limit of quantification for the established TRFIA-DON test strip.

**Concentration values (μg/kg)**				**Average**	**SD**	**LOQ (μg/kg)**
19.07	18.67	18.55	19.83	19.46	0.87	28.16
19.88	19.83	19.97	21.20			
19.02	20.57	19.60	19.80			
19.08	17.57	20.29	20.17			
18.55	19.40	20.57	17.57			

#### 3.6.2. Precision

We evaluated the precision of the DON strips by using three artificial DON contamination samples with concentrations of 100, 1,000 and 35,00 μg/kg, respectively. Results showed that the intra-batch CVs of the strips at the concentrations of 100, 1,000 and 35,00 μg/kg were 5.00, 3.12, and 4.67%, respectively, and the inter-batch CVs at those concentrations were 4.55, 6.60, and 4.24%, respectively (Table 4).

**Table 4:** Coefficient of variation for the established TRFIA-DON test strip.

**Batches**	**Items**	**100 μg/kg**	**1,000 μg/kg**	**3,500 μg/kg**
Intra-batch	Average	98.91	992.93	3,775.03
	SD	4.95	30.98	176.41
	CV (%)	5.00	3.12	4.67
Inter-batch	Average	100.87	1,035.99	3,621.03
	SD	4.59	68.33	153.61
	CV (%)	4.55	6.60	4.24

#### 3.6.3. Accuracy

The quality control material DON in maize sample was used to evaluate the accuracy of TRFIA-DON test strip. As shown in Table 5, the recovery rates in maize samples ranged from 92 to 104%, which could meet the requirements of daily detection.

**Table 5:** Recovery rates of the TRFIA-DON test strip in corn samples.

**Items**	**Copy 1**	**Copy 2**	**Copy 3**	**Copy 4**	**Copy 5**	**Copy 6**
Reference value (μg/kg)	1,410 ± 110	1,410 ± 110	1,410 ± 110	1,410 ± 110	1,410 ± 110	1,410 ± 110
Detection value (μg/kg)	1,254	1,464	1,383	1,356	1,411	1,349
Recovery rates	92%	104%	98%	96%	100%	96%
Average recovery rates	98%					

#### 3.6.4. Stability

**Figure 6:**
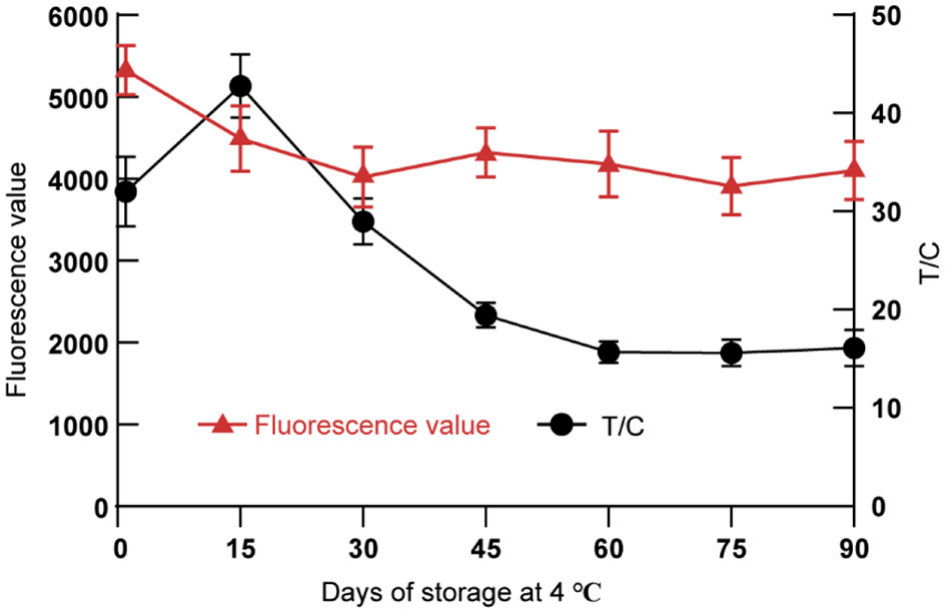
Stability of the TRFIA-DON test strip.

Through long-term storage (90 days) and random sampling test experiment at irregular intervals, we found that the fluorescence signals of T-line and C-line of DON test strips gradually tended to be stable after 60 days of storage at 4°C (Figure 6). The T/C values were used to calculate the CVs of the test strips with different storage times. The results showed that the CVs of the DON test strips on the 60th, 75th and 90th days was <5%.

#### 3.6.5. Applicability

To evaluate the applicability of developed TRFIA-DON test strips, a batch of real samples were tested by DON strips and LC-MS/MS, and the testing results were compared. Results showed that the relative standard deviation (RSD) between the DON strips and LC-MS/MS was <9% (Table 6). The DON in the real samples could be determined accurately and reliably *via* the established TRFIA-DON test strips. The DON test strips could meet the requirements of DON rapid detection in food crops and feeds.

**Table 6:** Comparison of TRFIA-DON test strip and LC-MS/MS for DON detection in crops and feeds.

**Samples**	**TRFIA (μg/kg)**	**LC-MS/MS (μg/kg)**	**RSD (%)**
LHF 1–10	301–2,426	318–2,651	1.12–6.66
PigF 1–10	349–1,999	338–2,018	0.12–7.58
PouF 1–10	523–3,926	590–4,000	1.50–6.92
PCF 1–10	607–2,650	540–2,710	1.24–6.00
SF 1–10	548–2,095	590–2,000	2.50–8.13
SFCC 1, 3, 5, 6	ND	ND	
SFCC 2, 4, 7–10	343–3,080	370–2,895	1.88–7.44
Wheat 1, 9, 10	ND	ND	
Wheat 2–8	648–2,895	660–2,822	1.12–3.27
Corn 2, 3, 6, 8	102–1,133	93–1,241	0.79–6.41
Corn 1, 4, 5, 7, 9, 10	ND	ND	
Flour 1, 2, 4–6, 8–10	110–794	103–750	0.88–6.01
Flour 3,7	ND	ND	

PouF, poultry feed; LHF, laying hen feed; PigF, piglet feed; PCF, pig compound feed; SF, sow feed; SFCC, supplemental feed for calf concentrates; RSD, relative standard deviation; ND, not detected.

## 4. Discussion

DON contamination is greatly affected by temperature and humidity, especially the temperature and humidity during harvest can directly affect the content and over-standard rate of DON in corn, wheat and other food crops. Corn, wheat, and soy are the main raw materials of livestock and poultry feed. The quality of feedstuff can significantly affect the early development of intestine and the microflora of animals, which in turn affect their growth performance (35, 36). Animals fed DON contaminated feed will appear vomiting, decreased feed intake, growth retardation and other symptoms (37). At the same time, DON can also be transferred into animal food such as milk, meat and eggs through feed, so as to be ingested by human body, posing a serious threat to human health (7). The fixed location and extensive scope of food crop harvesting increase the difficulty of market monitoring and supervision. In addition, more and more severe environmental conditions have posed new challenges to crop storage and the health of domestic animals (38, 39). Therefore, a simple, rapid, and accurate detection method is urgently needed for the detection of DON in food crops and feeds. Compared with the existing detection technology, the DON quantitative test strip established in this study has the advantages of strong specificity, high sensitivity, precise quantification and simple operation that can be used for both on-site and laboratory detection.

How to control and optimize the key factors such as the specificity of antibody and the quality of fluorescent microspheres affecting the performance of test strips is the difficulty and key point of developing rapidly quantitative test strips based on TRFIA technology (40). In the present study, we determined the optimal ratio of microspheres to antibodies (1:1), the optimal drying time of NC membrane (24 h) and other key factors affecting the performance of TRFIA-DON test strips. The LOQ of developed DON test strips is 28.16 μg/kg. Recently, a number of DON detection assays have been developed based on lateral-flow immunochromatographic (41), surface plasmon resonance (42), nanogold immunochromatographic (43), HPLC-UV technique (44), and TRFIA technology (31). The sensitivity of DON strips developed in this study is slightly higher than or close to that of those reported detection assays. It takes about 6 min to test food crop and feed samples with this paper strip, which is faster than recently developed nanogold immunochromatographic assay (45) and traditional LC-MS/MS. Compared with GICA and enzyme-linked immunosorbent assay (ELISA), TRFIA-DON test strip has the advantages of simple operation, good repeatability, and short detection time (Table 7). In addition, the results detected by this developed DON strips are nearly consistent with the LC-MS/MS detection results. These results show that the paper strip has high accuracy and reliability.

**Table 7:** Performance comparison of commonly used DON detection methods and TRFIA.

**Method performance**	**GICA**	**ELISA**	**LC-MS/MS**	**TRFIA**
Sensitivity	Low	Medium	High	High
Precision	Low	Medium	High	High
Quantitative	No	Semi-quantification	Yes	Yes
Testing time	≤15 min	≥60 min	>120 min	≤6 min
Testing procedure	Simplicity	Complex	Extremely complicated	Simplicity
Environmental requirement	Low	High	Extremely high	Low
Cost	Low	Medium	High	Low

## 5. Conclusion

DON contamination is one of the major global challenges to food and feed safety. Industry requires rapid, accurate, reliable, and portable testing methods to manage this challenge. In this study, we combined time-resolved fluorescence with immunochromatography to develop a TRFIA-DON test strip with the characteristics of high sensitivity, high accuracy, and a wide linear range. The accuracy of TRFIA-DON test strip in the detection of real food and feed samples was almost consistent with that of LC-MS/MS. This detection method is easy to operate and does not require dedicated facilities and highly qualified personnel. Moreover, the established TRFIA-DON test strip can rapid and quantitative determination of DON in food crops and feeds, which is applicable to the quantitative determination of DON at both on-site and laboratory.

## Data availability statement

The original contributions presented in the study are included in the article/supplementary material, further inquiries can be directed to the corresponding authors.

## Author contributions

JW, YZ, and LW contributed to conception and design of the study. JW, JL, LQ, and XM performed the experimental research and statistical analysis. JW wrote the first draft of the manuscript. JL, HZ, and XM wrote sections of the manuscript. YZ, LW, and HZ reviewed and edited the manuscript. All authors contributed to manuscript revision, read, and approved the submitted version.
